# Oxime-Based Carbonates as Useful Reagents for Both *N-*Protection and Peptide Coupling

**DOI:** 10.3390/molecules171214361

**Published:** 2012-12-05

**Authors:** Yahya El-Sayed Jad, Sherine N. Khattab, Ayman El-Faham, Fernando Albericio

**Affiliations:** 1 Department of Chemistry, Faculty of Science, Alexandria University, P.O. Box 426, Ibrahimia, 21321 Alexandria, Egypt; 2 Department of Chemistry, College of Science, King Saud University, P.O. Box 2455, 11451 Riyadh, Saudi Arabia; 3 Department of Chemistry and Molecular Pharmacology, Institute for Research in Biomedicine, Barcelona Science Park, Baldiri Reixac 10, 08028 Barcelona, Spain; 4 CIBER-BBN, Networking Centre on Bioengineering, Biomaterials and Nanomedicine, Barcelona Science Park, Baldiri Reixac 10, 08028 Barcelona, Spain; 5 Department of Organic Chemistry, University of Barcelona, Martí i Franqués 1-11, 08028 Barcelona, Spain; 6 School of Chemistry, University of KwaZulu-Natal, 4041 Durban, South Africa

**Keywords:** oxime, additives, *N*-protection, activation, coupling, peptide

## Abstract

We have demonstrated that oxime-based mixed carbonates are very effective reagents for both *N-*protection and peptide coupling.

## 1. Introduction

Peptides are increasingly gaining recognition as potential bioactive ingredients in the pharmaceutical industry [1,2,3]. Peptide synthesis depends on the strategies used for protecting the α-amino group and for activating the carboxylic acid group prior to peptide coupling. The two main classes [4,5,6] of carboxylic acid group activation methods are: (i) those that require *in situ* activation of the carboxylic acid and (ii) those that require an activated species that has previously been prepared (usually from an *in situ* activation step), isolated, purified, and characterized.

The amino group is most commonly protected by preparing the corresponding carbamate derivative. Despite the vast number of reagents reported to date for introducing the protecting group into the *N*-terminal amino group, there is still no universally active species capable of providing optimal protecting group introduction.

**Scheme 1:**
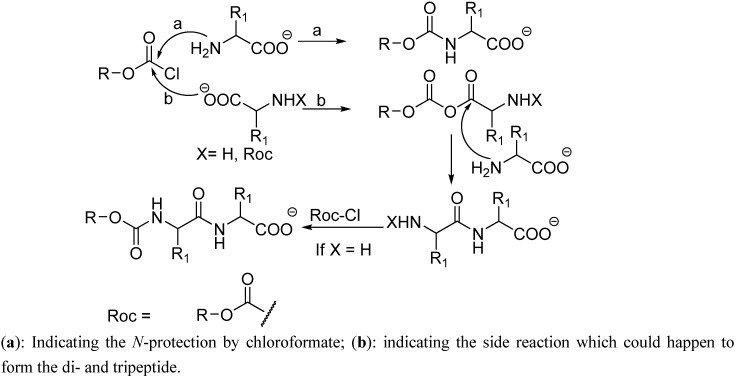
Mechanism for the formation side products (dipeptides and tripeptides) during the protection of amino acids with haloformates.

The traditional chloroformate strategy is an extremely powerful approach, providing fast amino group protection [7,8,9]. Nevertheless, in some cases, presence of the free carboxylic acid group can interfere with the reaction and lead to formation of byproducts such as dipeptides and even tripeptides (Scheme 1) [10].

**Figure 1:**
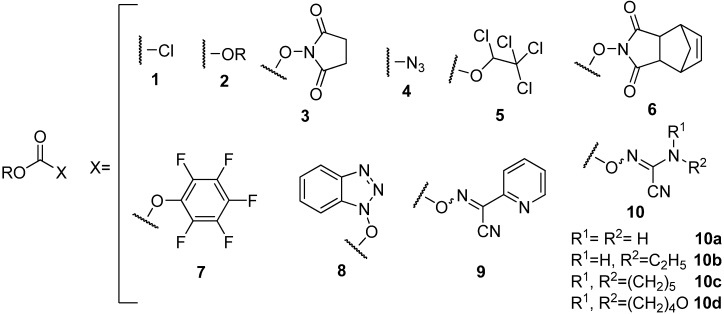
Structure of carbonates derivatives.

Since these side-reactions are associated with the quality of the leaving group, the less reactive species such as the dicarbonates **2** (Figure 1) [11,12,13,14] and the succinimidocarbonates **3** (Figure 1) [15,16] have previously been proposed as alternatives to the chloride **1 ** (Figure 1). Use of the azide derivatives **4** (Figure 1), [17,18] has also been proposed as an alternative for the chloride to prepare the *N*-protection of amino acids, but the explosive nature of azides precludes their use in large-scale synthesis. Moreover, several other approaches, based on the use of other less reactive species such as the 1,2,2,2-tetrachloroethyl [19,20], the 5-norbornene-2,3-dicarboximido [21], the pentafluorophenyl [22,23,24], and the 1-hydroxybenzotriazole [14,25,26] mixed carbonates **5**–**8** (Figure 1), have been proposed.

Recently, ethyl 2-cyano-2-(hydroxyimino)acetate (OxymaPure^®^, **12a**) has been tested as an additive for use in the carbodiimide approach for the formation of peptide bonds [27]. OxymaPure^®^ and its uronium-based phosphium coupling reagents displayed a remarkable capacity to inhibit racemization, together with impressive coupling efficiency, in both automated and manual synthesis, superior to those of **12d** and which has recently been reported to exhibit explosive properties [26] at least comparable to those of HOAt uronium-based phosphonium coupling reagents [28,29,30,31,32,33,34].

Later, we reported a series of Fmoc/Alloc-oxime carbonate reagents which are easy to prepare, stable, and highly reactive crystalline materials that afford nearly pure Fmoc/Alloc-amino acids in high yields. Among the Fmoc-oxime carbonates that we evaluated for the preparation of Fmoc/Alloc-Gly-OH, the *N-*hydroxypicolinimidoyl cyanide derivative **9** (Figure 1) gave the best results [35]. More recently, our research group reported the cyanoacetamide-based oximes **10** (Figure 1), which show unusual ability to afford Fmoc-protected amino acids in high yield, high purity and at lower cost relative to compound **9** [36].

Herein, we extended our studies for the synthesis of a new family of carbonate derivatives based on OxymaPure^®^, which are easy to prepare, stable, and have shown high efficiency in *N*-protection as well as peptide coupling.

## 2. Results and Discussion

### 2.1. Preparation of Carbonate Derivatives

**Scheme 2:**
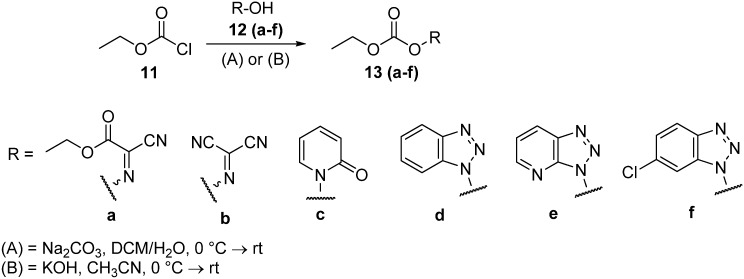
Preparation of the carbonate derivatives **13**.

The carbonate derivatives **13** were readily prepared by reacting ethyloxycarbonyl chloride (**11**) with an oxime (compounds **12a** or **12b**), *N*-hydroxy-2-pyridinone (**12c**) or benzotriazole derivatives (HOBt, **12d** or 6-Cl-HOBt, **12f**) in the presence of sodium carbonate in DCM/H_2_O (3:2) as solvent at 0 °C, with stirring at this temperature for 2 h (Scheme 2). After subsequent workup followed by isolation and recrystallization from CH_2_Cl_2_/hexane, ethyl 2-cyano-2-(ethoxycarbonyloxyimino)acetate (**13a)**, (ethoxycarbonyloxy)carbonimidoyl dicyanide (**13b**), ethyl 2-oxopyridin-1(2*H*)-yl carbonate (**13c**), 1*H*-benzo[*d*][1,2,3]triazol-1-yl ethyl carbonate (**13d**), and 6-chloro-1*H*-benzo[*d*][1,2,3]triazol-1-yl ethyl carbonate (**13f**), were prepared in 46–78% yield (Table 1).

**Table 1:** Yield, m.p. and elemental analysis of the carbonate derivatives **13**.

Compd.	Method	Yield (%)	m.p. (°C)	Elemental Analysis: Calculated (Found)		
				C	H	N
**13a**	A	78	44–45	44.86 (45.08)	4.71 (4.63)	13.08 (13.17)
**13b**	A	69	oily	43.12 (43.25)	3.02 (2.89)	25.14 (25.33)
**13c**	A	76	64–67	52.46 (52.21)	4.95 (5.16)	7.65 (7.91)
**13d**	A	52	138–139	52.17 (51.96)	4.38 (4.54)	20.28 (20.49)
13e *	B	77	133–135	46.16 (45.88)	3.87 (4.14)	26.91 (27.19)
**13f**	A	76	144–145	44.74 (44.53)	3.34 (3.61)	17.39 (17.18)

* **13e** was prepared by reacting ethyloxycarbonyl chloride **11** with HOAt **12e** in the presence of anhydrous potassium hydroxide (1 equivalent) in acetonitrile as solvent at 0 °C.

**Scheme 3:**
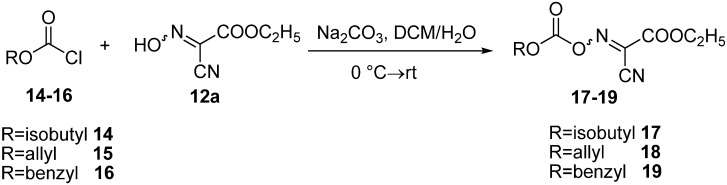
Preparation of oximinocarbonate derivatives.

Three different oxime carbonate derivatives: ethyl 2-cyano (isobutoxycarbonyloxyimino)acetate (**17**), ethyl 2-(allyloxycarbonyloxyimino)-2-cyanoacetate (**18**) and ethyl 2-(benzyloxycarbonyloxy-imino)-2-cyanoacetate (**19**) were prepared by reacting the corresponding chloroformates **14**–**16** and OxymaPure^®^ (**12a**) in the presence of sodium carbonate in DCM/H_2_O (3:2) as solvent at 0 °C (Scheme 3). After subsequent workup followed by isolation and recrystallization from CH_2_Cl_2_/hexane, the corresponding oxime carbonates **17**–**19** were obtained in 87–94% yield (Table 2).

**Table 2:** Yield, m.p. and elemental analysis of the oximinocarbonate derivatives **17**–**19**.

Product	Yield (%)	m.p. (°C)	Elemental Analysis: Calculated (Found)		
			C	H	N
**17**	93	59–60	49.58 (49.81)	5.83 (5.57)	11.56 (11.74)
**18**	94	Oily	47.79 (47.93)	4.46 (4.61)	12.39 (12.58)
**19**	87	99–100	56.52 (56.23)	4.38 (4.62)	10.14 (10.41)

### 2.2. Preparation of 4-(Ethoxycarbonylamino)benzoic Acid

**Scheme 4:**
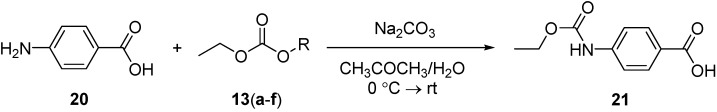
*N*-protection of 4-aminobenzoic acid using carbonate derivatives **13a**–**f**.

To study the reactivity of prepared carbonate derivatives **13a**–**f** and their utility for the preparation of the *N*-protected amino acids, we initiated our studies with 4-aminobenzoic acid (**20**), which on treatment with the previously synthesized carbonate derivatives **13a**–**f** in a homogenous acetone/aqueous solvent mixture in the presence of sodium carbonate with stirring overnight at room temperature, provides the product **21**. Samples of 4-(ethoxycarbonylamino)benzoic acid (**21**) were obtained from the different carbonate derivatives after removing the unreacted starting carbonate by extracting with ether and acidifying the aqueous layer with 1N HCl (Scheme 4). The purity of the product **21** was determined after injection onto reverse-phase HPLC are shown in Table 3.

**Table 3:** Yield %, m.p., purity % of 4-(ethoxycarbonylamino)benzoic acid **21**.

Carbonate	Yield (%)	m.p. (°C)	Purity * (%)
**13a**	43	198–200	95.3
**13b**	38	199–201	88.5
**13c**	59	198–200	100
**13d**	11	184–192	77.1
**13e**	31	198–202	97.0
**13f**	42	190–195	83.6

* The purity was determined by HPLC using the following Conditions: detection at 220 nm (Waters 996 PDA detector); Sunfire C_18_ column (3.5 µm 4.6 × 100 mm); linear gradient over 14 min (10 to 100% CH_3_CN in H_2_O/0.1% TFA); flow rate 1.0 mL/min. *t*_R_ [4-(ethoxycarbonylamino)benzoic acid] = 4.18 min.

Table 3 showed that, 1*H*-benzo[*d*][1,2,3]triazol-1-yl ethyl carbonate (**13d**) provided the lowest purity (77.1%) and yield (11%) of all the ethoxycarbonyl carbonate derivatives. (Ethoxycarbonyloxy)carbonimidoyl dicyanide (**13b**) and 6-chloro-1*H*-benzo[*d*][1,2,3]triazol-1-yl ethyl carbonate (**13f**) provided moderate levels of purity (88.5% and 83.6%) and yield (38% and 42%). Ethyl 2-cyano-2-(ethoxycarbonyloxyimino)acetate (**13a**) and 3*H*-[1,2,3]triazolo[4,5-*b*]pyridin-3-yl ethyl carbonate (**13e**) provided high purity (95.3% and 97.0%, respectively) and moderate yield (43% and 31%), while, ethyl 2-oxopyridin-1(2*H*)-yl carbonate (**13c**) provided the highest yield (59%) with excellent purity (100%) as indicated from the HPLC traces.

### 2.3. HPLC Study of the Rate of Formation of the Active Ester

Before attempting simultaneous protection and activation of amino acids, we tried to prepare active esters of *N*-protected amino acids using the carbonate derivatives **13a**, **17**, **18**, and **19** to ensure that these compounds can activate carboxylic acids by forming the corresponding active ester for different Fmoc-amino acids. The reaction of Fmoc-amino acids with oxime carbonate derivatives was monitored by HPLC to study the rate of formation of the active ester. Aliquots (5 µL) of the reaction mixture were taken, diluted with acetonitrile, and then analyzed by HPLC. Follow-up samples were studied at intervals of time 30 min and 1, 2, 4 and 24 h pre-activation. This enabled us to determine the optimum pre-activation time for each carbonate reagent, as excessively long times could lead to greater formation of alkyl or aryl esters.

#### 2.3.1. The Rate of Formation of the Active Ester of Fmoc-Val-OH Using Oxime Carbonate Derivatives

Mixing of Fmoc-Val-OH **22** with the oxime carbonate reagents EtocOXY **13a**, *^i^*BuocOXY **17**, AllocOXY **18** and ZOXY **19** in the presence of pyridine in DMF, we observed maximum levels of the active ester was formed at 4 h for **13a**, 2 h for **17**, 30 min for **18** and 1 h for **19**. Whereas the alkyl or aryl esters **26** started to be formed after 2 and 1 h in case of EtocOXY **13a** and *^i^*BuocOXY **17**, respectively, and after half an hour in case of AllocOXY **18** and ZOXY **19** respectively. Therefore, the optimum pre-activation time should not exceed more than 2 and 1 h in case of EtocOXY **13a** and *^i^*BuocOXY **17**, respectively, and should be less than half an hour in case of both AllocOXY **18** and ZOXY **19** systems. Best results for formation of the active ester were obtained with the oxime carbonate **17** and **18**, while the oxime carbonates **13a** and **19** gave high yield of the alkyl ester (Table 4, Table 5, Table 6 and Table 7).

**Table 4:** The rate of formation of the active ester of Fmoc-Val-OH **22** using ethyl 2-cyano-2-(ethoxycarbonyloxyimino)acetate (**13a**).

Pre-activation time (hr)	Oxyma 12a	Fmoc-Val-OH 22	Active ester 27	Ethyl ester
½	7.2	40.6	37.8	n/a
1	3.7	48.0	39.8	n/a
2	2.3	29.5	61.7	n/a
4	3.5	18.7	61.1	13.2
24	19.1	17.4	15.0	48.5

**Table 5:** The rate of formation of the active ester of Fmoc-Val-OH **22** using ethyl 2-cyano-2-(isobutoxycarbonyloxyimino)acetate (**17**).

Pre-activation time (hr)	Oxyma 12a	Fmoc-Val-OH 22	Active ester 27	Isobutyl ester
½	3.1	54.7	33.6	n/a
1	0.9	45.9	44.3	n/a
2	1.4	37.2	54.8	1.9
4	2.6	27.3	63.5	6.7
24	16.8	16.0	21.9	45.3

**Table 6:** The rate of formation of the active ester of Fmoc-Val-OH **22** usingethyl 2-(allyloxycarbonyloxyimino)-2-cyanoacetate (**18**).

Pre-activation time (hr)	Oxyma 12a	Fmoc-Val-OH 22	Active ester 27	Allyl ester
½	n/a	9.4	63.9	10.8
1	n/a	14.2	58.1	3.7
2	n/a	5.7	73.0	6.1
4	23.6	2.8	55.9	n/a

**Table 7:** The rate of formation of the active ester of Fmoc-Val-OH **22** using ethyl 2-(benzyloxycarbonyloxyimino)-2-cyanoacetate (**19**).

Pre-activation time (hr)	Oxyma 12a	Fmoc-Val-OH 22	Active ester 27	Benzyl ester
½	1.5	40.4	52.5	n/a
1	3.4	28.1	53.7	7.5
2	6.9	19.0	48.4	13.1
4	11.6	14.1	32.2	20.2
24	22.1	14.1	2.1	31.4

#### 2.3.2. The Rate of Formation of the Active Ester of Fmoc-Phe-OH Using Oxime Carbonate Derivatives

Due to the best results obtained from the previous example with the oxime carbonate derivatives **17** and **18**, Fmoc-Phe-OH **23** was tested with *^i^*BuocOXY **17** and AllocOXY **18** under the same conditions used in the previous example. From the results obtained from HPLC monitoring, the maximum levels of the active ester **27** are formed from the oxime carbonate derivatives **17** and **18** at 1 and 2 h, respectively; while the alkyl esters **26** appeared after 30 min and 1 hour, respectively. Thus, the pre-activation time in both systems should not exceed more than 30 min (Table 8 and Table 9).

**Table 8:** The rate of formation of the active ester of Fmoc-Phe-OH **23** using ethyl 2-cyano-2-(isobutoxycarbonyloxyimino)acetate (**17**).

Pre-activation time (hr)	Oxyma 12a	Fmoc-Phe-OH 23	Active ester 27	Isobutyl ester
½	n/a	28.3	43.0	14.7
1	0.8	15.8	72.0	8.4
2	0.5	19.5	58.2	6.1
4	0.8	16.2	63.3	1.5
24	3.5	21.4	52.7	22.0

**Table 9:** The rate of formation of the active ester of Fmoc-Phe-OH **23** using ethyl 2-(allyloxycarbonyloxyimino)-2-cyanoacetate (**18**).

Pre-activation time (hr)	Oxyma 12a	Fmoc-Phe-OH 23	Active ester 27	Allyl ester
½	4.7	24.7	58.56	
1	4.6	22.3	65.9	0.4
2	5.5	12.6	74.6	0.6
4	5.8	19.2	68.3	0.4
24	13.5	4.6	53.4	25.4

#### 2.3.3. The Rate of Formation of the Active Ester of Fmoc-Pro-OH Using Oxime Carbonate Derivatives

To ensure that we will get the same results with the oxime carbonate derivatives **17** and **18**, further study was performed with the more sterically hindered amino acid Fmoc-Pro-OH **24** using the two carbonate derivatives *^i^*BuocOXY **17** and AllocOXY **18**. From the results obtained by HPLC monitoring, we observed the maximum levels of the active ester **27** after 30 min and 2 h, respectively; while the alkyl esters **26** appeared after 30 min and 1 hour, respectively. Thus, the pre-activation time in both systems should not exceed more than 30 min, which in agreement with the previous results (Table 10 and Table 11).

**Table 10:** The rate of formation of the active ester of Fmoc-Pro-OH **24** using ethyl 2-Cyano-2-(isobutoxycarbonyloxyimino)acetate (**17**).

Pre-activation time (hr)	Oxyma 12a	Fmoc-Pro-OH 24	Active ester 27	Isobutyl ester
½	0.2	13.4	59.8	5.0
1	1.6	47.4	34.1	6.7
2	1.3	30.7	53.9	3.1
4	1.6	30.8	55.8	3.8
24	17.8	75.9	6.3	n/a

**Table 11:** The rate of formation of the active ester of Fmoc-Pro-OH **24** using ethyl 2-(allyloxycarbonyloxyimino)-2-cyanoacetate (**18**).

Pre-activation time (hr)	Oxyma 12a	Fmoc-Pro-OH 24	Active ester 27	Allyl ester
½	1.4	13.2	65.7	n/a
1	1.8	13.3	70.3	2.8
2	3.0	15.2	70.6	2.7
4	13.8	58.0	24.3	3.9
24	19.4	46.7	18.7	15.2

**Scheme 5:**
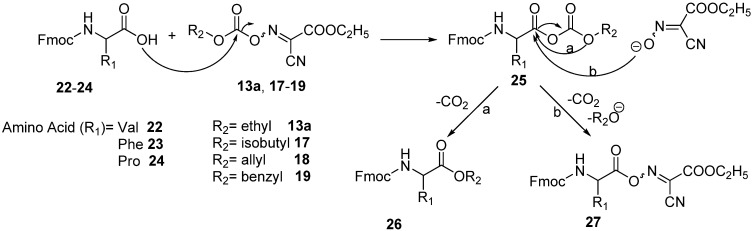
Mechanism of the formation of the oxime active ester using oxyme carbonates.

The results obtained from Table 4, Table 5, Table 6, Table 7, Table 8, Table 9, Table 10 and Table 11 may indicate that, the activation of carboxylic acid group of the amino acid using oximinocarbonate derivatives may proceed through a mixed anhydride-type intermediate **25**, which may react with the oxyma anion to afford the active ester **27** (Scheme 5). Its decarboxylation will afford alkyl or aryl esters **26**, which is a rather slow step. All intermediate stages of the reaction are run at low temperature to prevent side reactions.

### 2.4. Synthesis of Dipeptide Fmoc-Val-Ala-OMe

As an initial model to examine the reactivity of the oxime carbonate **13a**, **17**, **18** and **19** as coupling reagents, these reagents were examined in the stepwise coupling of a previously studied model system [29] Fmoc-Val-Ala-OMe **28**. The pre-activation time was determined for each of these coupling reagents according to the previous studies obtained from HPLC for the rate of formation of active ester. All coupling reactions were performed in the presence of 2 equiv. pyridine as a base and in DMF as solvent. The results for each coupling reagent are given in Table 12.

**Table 12:** Coupling of Fmoc-Val-OH with H-Ala-OMe **28** using different oxime carbonate derivatives.

Coupling reagent	Pre-activation time (hr)	m.p. (°C)	Yield (%)	HPLC Purity (%)
EtocOXY **13a**	2	195–196	77	100
*^i^*BuocOXY **17**	1	192–193	54	96.1
AllocOXY **18**	½	194–195	75	98.7
ZOXY **19**	½	194–195	64	97.7

The purity of the dipeptide **28** was determined by HPLC, and was found to be 96.1 to 100% at *t*_R LL_ [Fmoc-Val-Ala-OMe] = 8.32 min. From Table 12, the highest yield and purity was obtained with EtocOXY **13a**, while the lowest yield and purity was obtained with *^i^*BuocOXY **17** and ZOXY **19**. While, the AllocOXY **18** had moderate yield and purity. None of the D,L-isomer was detected by HPLC or NMR spectra in all the cases, but the impurities were related to the alkyl ester and oxime.

## 3. Experimental

### 3.1. Materials

The solvents used were of HPLC reagent grade. Melting points were determined with a Mel-Temp apparatus and are uncorrected. Infrared (IR) spectra were recorded on a Perkin-Elmer 1600 series Fourier transform instrument as KBr pellets. Nuclear Magnetic resonance spectra (^1^H-NMR and ^13^C-NMR spectra) were recorded on a JOEL 500 MHz and on a Mercury 400 MHz spectrometer with chemical shift values reported in δ units (ppm) relative to an internal standard. Elemental analyses were performed on Perkin-Elmer 2400 elemental analyzer, and the values found were within ±0.3% of the theoretical values. Follow-up of the reactions and checks of the purity of the compounds was done by TLC on silica gel-protected aluminum sheets (Type 60 GF254, Merck, Barcelona, Spain) and the spots were detected by exposure to UV-lamp at λ 254 nm for a few seconds. The compounds were named using ChemDraw Ultra version 11, CambridgeSoft Corporation (Cambridge, MA, USA).

### 3.2. General Method for Preparation of Ethyloxycarbonyl Derivatives ***13****(**a–d***, ***f**)*

A solution of ethyloxycarbonyl chloride (**11**, 0.95 mL, 10 mmol) in CH_2_Cl_2_ (30 mL) was added slowly to a solution of sodium carbonate (2.12 g, 20 mmol) and 10 mmol of oximes (**12a**, **12b**), 1-hydroxypyridin-2(1*H*)-one (**12c**), or benzotriazole derivatives (**12d** or **12f**) in H_2_O (20 mL) with stirring at 0 °C. The resulting clear mixture was stirred at 0°C for 30 min and then at room temperature for 2 h. After dilution with CH_2_Cl_2_ (50 mL), the organic phase was collected and washed with water and saturated aqueous NaCl (30 mL), dried over anhydrous Na_2_SO_4_ and then filtered, and the solvent was then removed on a rotary evaporator. The residue was recrystallized from CH_2_Cl_2_/hexane to give the ethyloxycarbonyl derivatives **13**(**a**–**d**, **f**).

*Ethyl 2-cyano-2-(ethoxycarbonyloxyimino)acetate* (**13a**). The product was obtained as white crystals (1.67 g; 78.17% yield) (m.p. 44–45 °C). IR (KBr): 2241 (w, CN), 1812 (s, CO), 1741 (s, CO, ester) cm^−1^. ^1^H-NMR (CDCl_3_): δ 1.39–1.41 (m, 6H, 2 CH_3_), 4.43–4.47 (m, 4H, 2 CH_2_). ^13^C-NMR (CDCl_3_): δ 14.14, 14.26, 64.76, 67.33, 106.67, 131.03, 150.87, 156.81. Elemental analysis Calcd for C_8_H_10_N_2_O_5_: C, 44.86; H, 4.71; N, 13.08. Found: C, 45.08; H, 4.63; N, 13.17.

*(Ethoxycarbonyloxy)carbonimidoyl dicyanide* (**13b**). The product was obtained as an oil at room temperature (1.15 g; 68.89% yield). IR (KBr): 2248 (w, CN), 1811 (s, CO, ester) cm^−1^. ^1^H-NMR (CDCl_3_): δ 1.43 (t, 3H, ^3^*J* = 7.2 Hz, CH_3_), 4.49 (q, 2H, ^3^*J* = 7.2 Hz, CH_2_). ^13^C-NMR (CDCl_3_): δ 14.18, 68.32, 104.81, 108.03, 114.44, 149.70. Elemental analysis: Calcd for C_6_H_5_N_3_O_3_: C, 43.12; H, 3.02; N, 25.14. Found: C, 43.25; H, 2.89; N, 25.33.

*Ethyl 2-oxopyridin-1(2H)-yl carbonate* (**13c**). The product was obtained as white crystals (1.38 g; 75.47% yield) (m.p. 64–67 °C). IR (KBr): 1792 (s, CO), 1668 (s, CO, amidic) cm^−1^. ^1^H-NMR (CDCl_3_): δ 1.42 (t, 3H, ^3^*J* = 7.2 Hz, CH_3_), 4.42 (q, 2H, ^3^*J* = 7.2 Hz, CH_2_), 6.20 (td, 1H, ^3^*J* = 6.8 Hz, ^4^*J* = 1.6 Hz, Py-H), 6.72–6.74 (m, 1H, Py-H), 7.36 (td, 1H, ^3^*J* = 6.8 Hz, ^4^*J* = 2 Hz, Py-H), 7.46 (dd, 1H, ^3^*J* = 6.8 Hz, ^4^*J* = 2 Hz, Py-H). ^13^C-NMR (CDCl_3_): δ 14.25, 67.53, 105.29, 123.18, 135.14, 139.69, 152.45, 157.31.

*1H-Benzo[d][1,2,3]triazol-1-yl ethyl carbonate* (**13d**). The product was obtained as white crystals (1.07 g; 51.84% yield) (m.p. 138–139 °C). IR (KBr): 1751 (s, CO) cm^−1^. ^1^H-NMR (CDCl_3_): δ 1.53 (t, 3H, ^3^*J* = 7.2 Hz, CH_3_), 4.63 (q, 2H, ^3^*J* = 7.2 Hz, CH_2_), 7.56 (td, 1H, ^3^*J* = 8.4 Hz, ^4^*J* = 0.8 Hz, Ar-H), 7.78 (td, 1H, ^3^*J* = 8.4 Hz, ^4^*J* = 1.2 Hz, Ar-H), 8.00 (d, 1H, ^3^*J* = 8.4 Hz, Ar-H), 8.21 (d, 1H, ^3^*J* = 8.4 Hz, Ar-H). ^13^C-NMR (CDCl_3_): δ = 14.42, 65.67, 115.27, 115.88, 126.46, 132.91, 133.54, 147.52. Elemental analysis: Calcd for C_9_H_9_N_3_O_3_: C, 52.17; H, 4.38; N, 20.28. Found: C, 51.96; H, 4.54; N, 20.49.

*6-Chloro-1H-benzo[d][1,2,3]triazol-1-yl ethyl carbonate* (**13f**). The product was obtained as white crystals (1.81 g; 75.67% yield) (m.p. 144–145 °C). IR (KBr): 1743 (s, CO) cm^−1^. ^1^H-NMR (CDCl_3_): δ 1.53 (t, 3H, ^3^*J* = 7.2 Hz, CH_3_), 4.62 (q, 2H, ^3^*J* = 7.2 Hz, CH_2_), 7.72 (dd, 1H, ^3^*J* = 8.8 Hz, ^4^*J* = 2 Hz, Ar-H), 8.00 (d, 1H, ^4^*J* = 2 Hz, Ar-H), 8.16 (d, 1H, ^3^*J* = 8.8 Hz, Ar-H). ^13^C-NMR (CDCl_3_): δ 14.42, 65.99, 115.70, 116.32, 132.19, 132.81, 133.78, 147.32. Elemental analysis: Calcd for C_9_H_8_ClN_3_O_3_: C, 44.74; H, 3.34; N, 17.39. Found: C, 44.53; H, 3.61; N, 17.18.

### 3.3. 3H-[1,2,3]Triazolo[4,5-b]pyridin-3-yl Ethyl Carbonate *(**13e**)*

A solution of HOAt (**12e**, 0.68 g, 5 mmol) and anhydrous potassium hydroxide (0.3 g, 5.5 mmol) in acetonitrile (5 mL) was cooled to 0 °C. A solution of ethyloxycarbonyl chloride (**11**, 0.47 mL, 5 mmol) in acetonitrile (5 mL) was slowly added dropwise for 30 min to the solution as it was stirred magnetically. The resulting clear mixture was stirred at room temperature overnight. It was then filtered, and the solvent was removed with a rotary evaporator. The residue was recrystallized from CH_2_Cl_2_/hexane to give 3*H*-[1,2,3]triazolo[4,5-*b*]pyridin-3-yl ethyl carbonate (**13e**). The product was obtained as 0.8 g (77.14% yield) of white crystals (m.p. 133–135 °C). IR (KBr): 1744 (s, CO) cm^−1^. ^1^H-NMR (DMSO): δ 1.05 (t, 3H, ^3^*J* = 7.2 Hz, CH_3_), 3.43 (q, 2H, ^3^*J* = 7.2 Hz, CH_2_), 7.49–7.53 (m, 1H, Ar-H), 8.52–8.55 (m, 1H, Ar-H), 8.75–8.77 (m, 1H, Ar-H), ^13^C-NMR (DMSO): δ 18.47, 55.94, 120.62, 124.09, 134.52, 139.50, 151.01. Elemental analysis: Calcd for C_8_H_8_N_4_O_3_: C, 46.16; H, 3.87; N, 26.91. Found: C, 45.88; H, 4.14; N, 27.19.

### 3.4. General Method for Preparation of Oxime Carbonate Derivatives ***17–19***

A solution of chloroformate (10 mmol) [isobutyloxycarbonyl chloride (**14**), allyloxycarbonyl chloride (**15**) or benzyloxycarbonyl chloride (**16**)] in CH_2_Cl_2_ (30 mL) was added slowly to a solution (10 mmol) of oxima **12a** and sodium carbonate (2.12 g, 20 mmol) in H_2_O (20 mL) with stirring at 0 °C. The resulting clear mixture was stirred at 0 °C for 30 min and then at room temperature for 2 h. After dilution with CH_2_Cl_2_ (50 mL), the organic phase was collected, washed with water and saturated aqueous NaCl (30 mL), and then dried over anhydrous MgSO_4_. It was then filtered, and the solvent was removed with a rotary evaporator. The residue was recrystallized from CH_2_Cl_2_/hexane to give oxime carbonate derivatives **17**–**19**.

*Ethyl 2-cyano-2-(isobutoxycarbonyloxyimino)acetate* (**17**). The product was obtained as a white solid (2.42 g; 93% yield) (m.p. 59–60 °C). IR (KBr): 1814 (s, CO), 1758 (s, CO, ester) cm^−1^. ^1^H-NMR (CDCl_3_): δ 1.00 (d, *J* = 6.8 Hz, 6H, 2 CH_3_), 1.42 (t, *J* = 7.2 Hz, 3H, CH_3_), 2.06–2.13 (m, 1H, CH), 4.17 (d, *J* = 6.4 Hz, 2H, CH_2_), 4.50 (q, *J* = 7.2 Hz, 2H, CH_2_). ^13^C-NMR (CDCl_3_): δ 14.16, 18.89, 27.95, 64.75, 106.70, 130.97, 151.10, 156.84. Elemental analysis: Calcd for C_10_H_14_N_2_O_5_: C, 49.58; H, 5.83; N, 11.56. Found: C, 49.81; H, 5.57; N, 11.74. The purity of **17** was determined after injection onto reverse-phase HPLC. Conditions: detection at 254 nm Waters 996 PDA detector, Sunfire C_18_ column 3.5 µm 4.6 ° 100 mm, linear gradient over 14 min of 10 to 100% CH_3_CN in H_2_O/0.1% TFA, flow rate 1.0 mL/min. *t*_R_ [ethyl 2-cyano-2-(isobutoxycarbonyloxyimino)acetate] = 7.38 min; purity 100%.

*Ethyl 2-(allyloxycarbonyloxyimino)-2-cyanoacetate* (**18**). The product was obtained as an oily substance that solidified in the refrigerator (2.26 g; 94% yield). IR (KBr): 2211 (w, CN), 1809 (s, CO), 1758 (s, CO, ester) cm^−1^. ^1^H-NMR (CDCl_3_): δ 1.42 (t, 3H, ^3^*J* = 7.2 Hz, CH_3_), 4.48 (q, 2H, ^3^*J* = 7.2 Hz, CH_2_), 4.85–4.87 (m, 2H, CH_2_), 5.39–5.51 (m, 2H, CH_2_), 5.96–6.03 (m, 1H, CH). ^13^C-NMR (CDCl_3_): δ 14.11, 64.78, 71.23, 106.62, 121.27, 130.07, 131.24, 150.74, 156.73. Elemental analysis. Calcd for C_9_H_10_N_2_O_5_: C, 47.79; H, 4.46; N, 12.39. Found: C, 47.93; H, 4.61; N, 12.58. The purity of **18** was determined after injection onto reverse-phase HPLC. Conditions: detection at 254 nm Waters 996 PDA detector, Sunfire C_18_ column 3.5 µm 4.6 ° 100 mm, linear gradient over 14 min of 10 to 100% CH_3_CN in H_2_O/0.1% TFA, flow rate 1.0 mL/min. *t*_R_ [ethyl 2-(allyloxycarbonyloxyimino)-2-cyanoacetate] = 6.69 min; purity 100%.

*Ethyl 2-(benzyloxycarbonyloxyimino)-2-cyanoacetate* (**19**). The product was obtained as white crystals (2.76 g; 87% yield) (m.p. 99–100 °C). IR (KBr): 1802 (s, CO), 1743 (s, CO, ester) cm^−1^. ^1^H-NMR (CDCl_3_): δ 1.41 (t, 3H, ^3^*J* = 7.2 Hz, CH_3_), 4.47 (q, 2H, ^3^*J* = 7.2 Hz, CH_2_), 5.38 (s, 2H, CH_2_), 7.40–7.44 (m, 5H, Ar-H). ^13^C-NMR (CDCl_3_): δ 14.17, 64.80, 72.57, 106.63, 129.06, 129.23, 129.63, 131.23, 133.59, 151.00, 156.76. Elemental analysis: Calcd for C_13_H_12_N_2_O_5_: C, 56.52; H, 4.38; N, 10.14. Found: C, 56.23; H, 4.62; N, 10.41. The purity of **19** was determined after injection onto reverse-phase HPLC. Conditions: detection at 254 nm Waters 996 PDA detector, Sunfire C_18_ column 3.5 µm 4.6 ° 100 mm, linear gradient over 14 min of 10 to 100% CH_3_CN in H_2_O/0.1% TFA, flow rate 1.0 mL/min. *t*_R_ [ethyl 2-(benzyloxycarbonyloxyimino)-2-cyanoacetate] = 7.31 min; purity 100%.

### 3.5. Synthesis of 4-(Ethoxycarbonylamino)benzoic Acid *(**21**)*

A solution of ethyloxycarbonyl derivative **13**(**a**–**f**) (1 mmol) in acetone (10 mL) was added dropwise to a stirring solution of 4-aminobenzoic acid **20** (0.14 g, 1 mmol) and sodium carbonate (0.32 g, 3 mmol) in acetone (20 mL) and H_2_O (10 mL). After stirring overnight, the reaction mixture was concentrated under reduced pressure, and then extracted with CH_2_Cl_2_ (20 mL) to remove the unreacted ethyloxycarbonyl derivatives. The reaction mixture was acidified with 1 N HCl (detected with Congo red litmus paper) to give a white solid, which was filtered, washed with water several times, dried and then recrystallized (ethyl acetate/*n*-hexane) to give a white solid. The purity of **21** was determined by reverse-phase HPLC. Conditions: detection at 220 nm (Waters 996 PDA detector); Sunfire C_18_ column (3.5 µm 4.6 × 100 mm); linear gradient over 14 min (10 to 100% CH_3_CN in H_2_O/0.1% TFA); flow rate 1.0 mL/min. *t*_R_ [4-(ethoxycarbonylamino)benzoic acid] = 4.18 min. IR (KBr): 3334 (w, NH), 3400–2500 (br, OH, acid), 1704 (s, CO, acidic), 1686 (s, CON) cm^−1^. ^1^H-NMR (DMSO): δ 1.24 (t, 3H, ^3^*J* = 7.2 Hz, CH_3_), 4.13 (q, 2H, ^3^*J* = 7.2 Hz, CH_2_), 7.54 (d, 2H, ^3^*J* = 8.4 Hz, Ar-H), 7.82 (d, 2H, ^3^*J* = 8.4 Hz, Ar-H), 9.93 (s, 1H, NH). ^13^C-NMR (DMSO): δ 15.11, 61.11, 117.91, 124.91, 131.05, 144.12, 153.00, 167.63.

### 3.6. HPLC Study of the Rate of Formation of Active Esters

#### 3.6.1. The Rate of Formation of the Active Ester of Fmoc-Val-OH **22** Using Oxime Carbonate Derivatives **13a**, **17–19**

A solution of Fmoc-Val-OH **22** (0.0423 g, 0.125 mmol) and the oxime carbonate derivatives **13a**, **17**, **18** or **19** (0.125 mmol) was dissolved in DMF (2 mL) in the presence of pyridine (20 µL). The reaction was monitored by HPLC. Aliquots (5 µL) were taken from the reaction mixture, diluted with ACN and detected by HPLC. Follow-ups were done at 30 min and at 1, 2, 4 and 24 h pre-activation. The percentages of OxymaPure^®^ **12a**, Fmoc-Val-OH **22**, active ester **27**, and alkyl or aryl ester **26** were determined by HPLC analysis of the diluted reaction mixture. Conditions: detection at 254 nm (Waters 996 PDA detector); Sunfire C_18_ column (3.5 µm 4.6 × 100 mm); linear gradient over 14 min (10 to 100% CH_3_CN in H_2_O/0.1% TFA); flow rate 1.0 mL/min. *t*_R_ [active ester] = 8.4 min. The percentages of OxymaPure^®^ **12a**, Fmoc-Val-OH **22**, active ester **27**, and alkyl or aryl esters **26** are shown in Table 4, Table 5, Table 6 and Table 7.

#### 3.6.2. The Rate of Formation of the Active Ester of Fmoc-Phe-OH 23 Using Oxime Carbonate Derivatives **17** or **18**

A solution of Fmoc-Phe-OH **23** (0.0483 g, 0.125 mmol) and oxime carbonate derivative **17** or **18** (0.125 mmol) was dissolved in DMF (2 mL) in the presence of pyridine (20 µL). The reaction was monitored by HPLC. Aliquots (5 µL) were taken from the reaction mixture, diluted with ACN and detected by HPLC. Follow-ups were done at 30 min and at 1, 2, 4 and 24 h pre-activation. The percentages of OxymaPure^®^ (**12a**), Fmoc-Phe-OH **23**, active ester **27** and alkyl ester **26** were determined by HPLC analysis of the diluted reaction mixture. Conditions: detection at 254 nm (Waters 996 PDA detector); Sunfire C_18_ column (3.5 µm 4.6 × 100 mm); linear gradient over 14 min (10 to 100% CH_3_CN in H_2_O/0.1% TFA); flow rate 1.0 mL/min. *t*_R_ [active ester] = 8.4 min. The percentages of OxymaPure^®^ (**12a**), Fmoc-Phe-OH **23**, active ester **27** and alkyl esters **26** are shown in Table 8 and Table 9.

#### 3.6.3. The Rate of Formation of the Active Ester of Fmoc-Pro-OH 24 Using Oxime Carbonate Derivatives **17** or **18**

A solution of Fmoc-Pro-OH **24** (0.0421 g, 0.125 mmol) and oxime carbonate derivative **17** or **18** (0.125 mmol) was dissolved in DMF (2 mL) in the presence of pyridine (20 µL). The reaction was monitored by HPLC. Aliquots (5 µL) were taken from the reaction mixture, diluted with ACN and analyzed by HPLC. Follow-ups were done at 30 min and at 1, 2, 4 and 24 h pre-activation. The percentages of OxymaPure^®^ (**12a**), Fmoc-Pro-OH **24**, active ester **27** and alkyl ester **26** were determined by HPLC analysis of the diluted reaction mixture. Conditions: detection at 254 nm Waters 996 PDA detector, Sunfire C_18_ column 3.5 µm 4.6 × 100 mm, linear gradient over 14 min of 10 to 100% CH_3_CN in H_2_O/0.1% TFA, flow rate 1.0 mL/min. *t*_R_ [active ester] = 8.2 min. The percent of Oxyma **12a**, Fmoc-Pro-OH **24**, active ester **27** and alkyl esters **26** are shown in Table 10 and Table 11.

### 3.7. General Method for the Synthesis of Dipeptide Fmoc-Val-Ala-OMe **28**

A solution of Fmoc-Val-OH **22** (0.339 g, 1 mmol) and the appropriate coupling reagent (1 mmol) in DMF (2 mL) was cooled to 0 °C and treated dropwise with pyridine (0.088 mL, 1.1 mmol). The reaction mixture was stirred for pre-activation at different times, depending on the conditions of the entry studied, and then treated with a solution of H-Ala-OMe.HCl (0.139 g, 1 mmol) and pyridine (0.088 mL, 1.1 mmol) in DMF (1 mL). The reaction mixture was stirred overnight. After dilution with 25 mL of ethyl acetate, the organic phase was washed with 5% citric acid (3 × 15 mL), saturated aq. NaHCO_3_ (3 × 15 mL) and saturated aq. NaCl (3 × 15 mL), and then dried over anhydrous Na_2_SO_4_ and filtered. The solvent was removed with a rotary evaporator, and the residue was recrystallized from CH_2_Cl_2_/hexane to give the dipeptide Fmoc-Val-Ala-OMe **28**. The purity of **28** was by reverse-phase HPLC. Conditions: detection at 220 nm (Agilent 1200 PDA detector); Eclipse plus C_18_ column (3.5 µm 4.6 × 100 mm); linear gradient over 14 min (10 to 100% CH_3_CN in H_2_O/0.1% TFA); flow rate 1.0 mL/min. *t*_R LL_ [Fmoc-Val-Ala-OMe] = 8.32 min. The results of coupling of Fmoc-Val-OH with H-Ala-OMe using different oximinocarbonate derivatives are shown in Table 12. ^1^H-NMR (CDCl_3_): δ 0.94–0.98 (m, 6H, 2CH_3_), 1.40 (d, 3H, ^3^*J* = 6.9 Hz, CH_3_), 2.10–2.11 (m, 1H, CH), 3.73 (s, 3H, CH_3_), 3.90–4.00 (m, 1H, CH), 4.17–4.21 (m, 1H, CH), 4.34–4.43 (m, 2H, CH_2_), 4.55–4.58 (m, 1H, CH), 5.47–5.51 (m, 1H, NH), 6.42–6.45 (m, 1H, NH), 7.25–776 (m, 8H, Ar-H).

## 4. Conclusions

Protection of the amino group and activation of the carboxylic acid groups are the most important steps associated with peptide synthesis. A possible strategy is to use oxime carbonate derivatives to simultaneously protect the amino group as a carbamate derivative and activate the carboxylic acid group as an active oxime ester was performed. A detailed study is carried out to understand the scope and limitations of this method using different oxime carbonate derivatives. The efficiency of these derivatives depends on the nature of oxime carbonates and also on the nature of the amino acids. From our studies we determined that the new family of oximes are useful reagents for both *N-*protection and activation of the protected amino acid. As a final conclusion from our studies, the *^i^*BuocOXY compound **17** and AllocOXY compound **18** both give the best results for formation of the active ester with less alkyl ester formation, while the EtocOXY compund **13a** gave the best results for the coupling step. The ZOXY reagent **19** might be not useful in either the activation or coupling steps.
